# 2015: A Transition Year for *Pharmaceuticals*?

**DOI:** 10.3390/ph9010006

**Published:** 2016-01-28

**Authors:** Jean Jacques Vanden Eynde

**Affiliations:** Editor-in-Chief, MDPI AG, Klybeckstrasse 64, Basel CH-4057, Switzerland; jean-jacques.vandeneynde@ex.umons.ac.be

In this period, I could not start this editorial without wishing all of you a Happy New Year.

This is also the opportunity for me to warmly thank, for their confidence, our authors, readers, reviewers, members of the editorial board, sponsors, as well as members of MDPI AG in Basel, Beijing, and Wuhan.

## Looking Back on 2015

When I was offered the position of Editor-in-Chief of *Pharmaceuticals* at the end of January, I was told that the job would be challenging and exciting. It was true, but I got a great deal of support from the scientific and administrative teams of MDPI AG. That allowed us to accumulate excellent results during the past year. Visibility of *Pharmaceuticals* increased from 458,383 full-text views in 2014 to 608,080 in 2015, a jump of almost 30%. Three Special Issues have been successfully completed. They were dedicated to mitochondrial target-based drug discovery [1] (Guest Editors: P. Hogg and P. Dilda), probiotics and prebiotics [2] (Guest Editor: Y. Koga), and microbial biofilms [3] (Guest Editor: D. Ren). A fourth Special Issue, entitled “Choices of the Journal” [4], has been launched and will remain open in 2016. It enables Guest Editors, as well as Editorial Board Members, to invite leading investigators to share their knowledge with our readers.

In 2015, eight distinguished scientists joined the Editorial Board: Jean Pierre Bazureau (Université de Rennes 1, Rennes, France), Tien L. Huang (Xavier University of Louisiana, New Orleans, LA, USA), Joachim Jose (Westfälische Wilhelms-Universität, Münster, Germany), Christopher W. K. Lam (Macau University of Science and Technology, Macau, China), Viktor P. Lozitsky (Research Center "Biomedical Testing of Preparations and Products", Odessa, Ukraine), Louis M. Mansky (University of Minnesota, Minneapolis, MN, USA), Suzanne Peyrottes (Université de Montpellier, Montpellier, France), and Maria Emilia de Sousa (Universidade do Porto, Porto, Portugal). Annie Mayence (formerly Haute Ecole Provinciale de Hainaut Condorcet, Saint-Ghislain, Belgium) acts as a new associate editor.

*Pharmaceuticals* has affirmed its status of international scientific journal by sponsoring a series of events among which:

- 23. Jahrestagung der Arbeitsgemeinschaft Radiochemie/Radiopharmazie, Erlangen, Germany;
- 29^èmes^ Journées Franco-Belges de Pharmacochimie, Spa, Belgium;
- 1st joint European Conference on Therapeutic Targets and Medicinal Chemistry, Münster, Germany;
- 23rd National Meeting on Medicinal Chemistry, Salerno, Italy;
- 11° Encontro Nacional de Quimica Organica/4° Encontro de Quimica Terapeutica, Porto, Portugal;
- Ricai 2015: 35th Interdisciplinary Meeting of Anti-infective Chemotherapy, Paris, France.

The journal was also present at the 250th American Chemical Society National Meeting and Exhibition in Boston, MA, USA. In addition, last but not least, *Pharmaceuticals* was the proud organizer of the First International Electronic Conference of Medicinal Chemistry [5]. That conference effectively deserves the adjective “international” since it gathered 206 authors from 18 different countries: Belgium, Bulgaria, Canada, France, Germany, India, Ireland, Italy, Japan, Poland, Portugal, Russia, Spain, Switzerland, Ukraine, United Kingdom, United States of America, and Uzbekistan. For its first edition, the conference can be considered a success. Indeed, the website was visited by more 25,000 individuals who could browse among the 55 presentations, videos, and keynotes. It also attracted 10 media partners; we are grateful to them for having widely publicized the event (Analis, Auriga, Eurisotop, Hielscher, Hitgen, Latoxan, Magritek, Reaction Biology Corp, Sairem, and Wylton). The award for the most viewed presentation during the conference was given to the group of C. Nienberg, A. Retterath, K.S. Becher, H.D. Mootz, and J. Jose for their work entitled “Click chemistry for advanced drug discovery applications of human protein kinase CK2” [6]. Members of the scientific advisory committee elected as the best presentation the work of K. Zhu and M. Kai, entitled “Convenient drug-resistance testing of HIV mutants” [7].

## Looking Forward to 2016

No time for napping; in 2016 we must confirm the promising results obtained during 2015. Increasing the visibility of our journal will remain one of our main goals. We are already working in that direction, and we are pleased to announce that the Second International Electronic Conference on Medicinal Chemistry [8] will take place in November. It is our pleasure to invite you to attend that event and to contribute to the growing success of *Pharmaceuticals*. We are also proud to let you know that the journal will organize a competition awarding one travel grant [9]. It will help a Ph.D. student or a young post-doctoral fellow to present a communication at an international meeting of her/his choice.

## References

[1] Hogg P., Dilda P. Mitochondrial Target-Based Drug Discovery.

[2] Koga Y. Probiotics and Prebiotics 2015.

[3] Ren D. Microbial Biofilms.

[4] Vanden Eynde J.J., Mayence A., Mineishi S. Choices of the Journal.

[5] The First International Electronic Conference on Medicinal Chemistry. http://sciforum.net/conference/ecmc-1.

[6] Click chemistry for advanced drug discovery applications of human protein kinase CK2. http://sciforum.net/conference/ecmc-1/paper/3138.

[7] Convenient drug-resistance testing of HIV mutants. http://sciforum.net/conference/ecmc-1/paper/3148.

[8] The Second International Electronic Conference on Medicinal Chemistry. http://sciforum.net/conference/ecmc-2.

[9] 2016 Travel Award. http://www.mdpi.com/journal/pharmaceuticals/awards.pdf.

